# Inhibition of CCL2 Signaling in Combination with Docetaxel Treatment Has Profound Inhibitory Effects on Prostate Cancer Growth in Bone

**DOI:** 10.3390/ijms140510483

**Published:** 2013-05-21

**Authors:** Peter S. Kirk, Theodore Koreckij, Holly M. Nguyen, Lisha G. Brown, Linda A. Snyder, Robert L. Vessella, Eva Corey

**Affiliations:** 1 Department of Urology, University of Washington, Seattle, WA 98195, USA; E-Mails: pstanfordkirk@gmail.com (P.S.K.); tkoreckij@gmail.com (T.K.); hmnguyen@uw.edu (H.M.N.); brown@uw.edu (L.G.B.); vessella@uw.edu (R.L.V.); 2 Department of Orthopedic Surgery, Wayne State University, Detroit, MI 48202, USA; 3 Janssen Research and Development, LLC, Spring House, PA 19002, USA; E-Mail: lsnyder2@its.jnj.com

**Keywords:** prostate cancer, bone metastases, chemokine, CCL2, docetaxel, bone metastases

## Abstract

The C-C chemokine ligand 2 (CCL2) stimulates migration, proliferation, and invasion of prostate cancer (PCa) cells, and its signaling also plays a role in the activation of osteoclasts. Therefore targeting CCL2 signaling in regulation of tumor progression in bone metastases is an area of intense research. The objective of our study was to investigate the efficacy of CCL2 blockade by neutralizing antibodies to inhibit the growth of PCa in bone. We used a preclinical model of cancer growth in the bone in which PCa C4-2B cells were injected directly into murine tibiae. Animals were treated for ten weeks with neutralizing anti-CCL2 antibodies, docetaxel, or a combination of both, and then followed an additional nine weeks. CCL2 blockade inhibited the growth of PCa in bone, with even more pronounced inhibition in combination with docetaxel. CCL2 blockade also resulted in increases in bone mineral density. Furthermore, our results showed that the tumor inhibition lasted even after discontinuation of the treatment. Our data provide compelling evidence that CCL2 blockade slows PCa growth in bone, both alone and in combination with docetaxel. These results support the continued investigations of CCL2 blockade as a treatment for advanced metastatic PCa.

## 1. Introduction

Although death rates for prostate cancer (PCa) have been in decline since the mid-1990s, PCa remains the second most common cause of cancer-related death in American men [1]. The initial treatment for advanced and/or metastatic PCa typically involves androgen deprivation therapy (ADT). However, most patients become refractory to ADT, developing castration-resistant disease (CRPC). The current standard of care for CRPC, docetaxel, offers a survival benefit of 2–3 months [2–5] and thus a great deal of emphasis continues to be placed on finding new improved therapies. Recently, multiple new treatments of advanced CRPC have been approved by the FDA that result in increased survival, but these treatments are not yet considered standard (e.g., abiraterone, sipuleucel-t, and MDV3100). Although previous strategies seeking improved therapies against advanced PCa focused specifically on targeting the tumor cell, current research is placing an emphasis on targeting the tumor microenvironment in addition to tumor cells. Chemokine signaling pathways are critically intertwined in the development and progression of PCa as well as in bone biology. Therefore, inhibition of chemokine signaling, which will not only affect tumor growth but also alter the bone microenvironment, is a promising prospective target for treatment of PCa bone metastases. CCL2 (also known as monocyte chemoattractant protein-1 or MCP-1), is a member of the C-C chemokine family, and its signaling cascade has received much interest as an important player in PCa.

CCL2 levels in patients with PCa bone metastases are increased over those in patients with primary PCa [6], and patients with a high Gleason score PCa (>7) and a high pathological classification (<pT3) exhibit significantly higher levels of CCL2, which in turn correlates significantly with biochemical recurrence [7]. Furthermore, three SNPs in CCL2 were found to be associated with high Gleason score (>7), one of which was associated with pathological staging, supporting a role of CCL2 in cancer development and progression [8].

In preclinical studies, CCL2 was shown to modulate invasion and apoptosis of PC-3 cells [9], and we have shown that knockdown of CCL2 in PC-3 cells leads to inhibition of their growth in tibiae of mice [6]. In a different study, over-expression of CCL2 in PC-3 cells increased proliferation and tumor growth *in vivo* as well as growth of PC-3 cells in the bone after cardiac injection, while inhibition of CCL2 signaling decreased growth of these cells [10]. In addition to tumor-promoting effects, CCL2 signaling is involved in regulation of the tumor microenvironment, playing a role in osteoclast and macrophage biology as well as angiogenesis [6,11–22]. Inhibition of CCL2 signaling affected osteoclastogenesis and infiltration of macrophages into PC-3 tumors [10]. Other treatment strategies targeting CCL2 signaling include a short peptide that mimics part of CCR2 (the CCL2 receptor) and neutralizes CCL2 activity, which inhibited growth of PC-3 subcutaneous tumors [23], and bindarit, a compound that inhibits CCL2 expression, which decreased PC-3 cell colonization of bone [24]. There are also reports of CCL2 blockade with neutralizing antibodies showing the efficacy of this treatment in PC-3 and VCaP tumors [11,22]. Multiple reviews summarize the importance and involvement of CCL2 in PCa progression and potential mechanisms of these effects [25–27].

The objective of our study was to evaluate the efficacy of CCL2 blockade and its combination with docetaxel to inhibit growth of PCa in the bone environment. Our results show that CCL2 blockade significantly inhibits tumor growth, and that docetaxel treatment augments this inhibition. Furthermore, we observed a sustained benefit of reduced tumor growth even after cessation of therapy. Our results also show effects of CCL2 blockade on the host response to tumor burden and systemic effects on normal bone.

## 2. Results and Discussion

### 2.1. CCL2 Blockade Inhibits Prostate Tumor Progression in Bone

**Figure 1:**
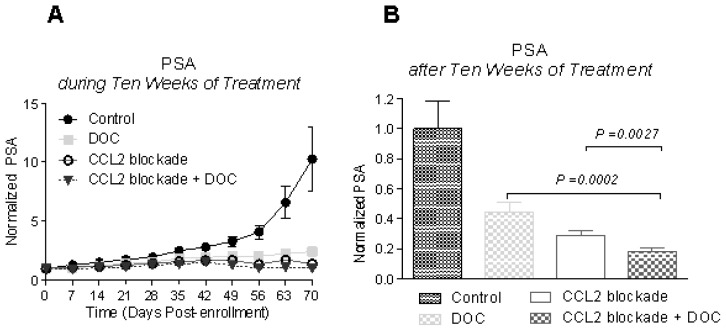

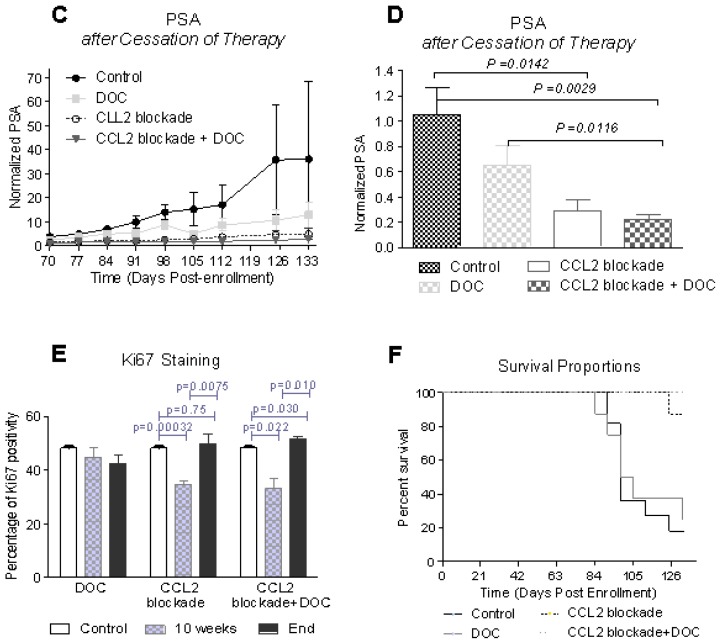
Effects of treatment on serum prostate-specific antigen (PSA), proliferation, and survival. Animals were injected with C4-2B cells directly into tibiae. Once tumors were established (based on serum PSA measurement), animals were randomized into treatment groups as described in the Methods Section. Serum PSA levels were normalized to enrollment values to minimize baseline differences. Normalized serum PSA levels plotted as mean ± SEM. (**A**) PSA response during the treatment period. Serum PSA levels were significantly lower in all treatment groups *vs.* control group starting at one week of treatment (*p* < 0.05); (**B**) Treatments resulted in significant differences in serum PSA levels at day 70 (control group set to 1); (**C**) After the treatments were stopped at day 70, a subset of animals was followed to evaluate any lasting effects of the treatments. Administration of CCL2 blockade and its combination with docetaxel showed sustained inhibition of tumor re-growth up to nine weeks following treatment termination; (**D**) The combination of CCL2 blockade and docetaxel had the most pronounced inhibitory effects on tumor growth as demonstrated by lowest levels of terminal serum PSA levels (control group set to 1); (**E**) Ki67 staining in untreated control tumors and treated tumors. Three areas of 4–6 samples per group were evaluated with 100–200 cells per area and percentage of positive cells was calculated. Decreased Ki67 positivity was detected in tumors treated with CCL2 blockade alone or in combination with docetaxel at the end of the treatment period, but positivity increased by the end of the follow up period, indicating future recurrence of the tumors; (**F**) Log-rank test of survival analysis shows significant survival benefits (CCL2: *p* = 0.0021, CCL2 + DOC: *p* = 0.0003) conferred by CCL2 blockade and its combination with docetaxel treatment.

**Figure 2:**
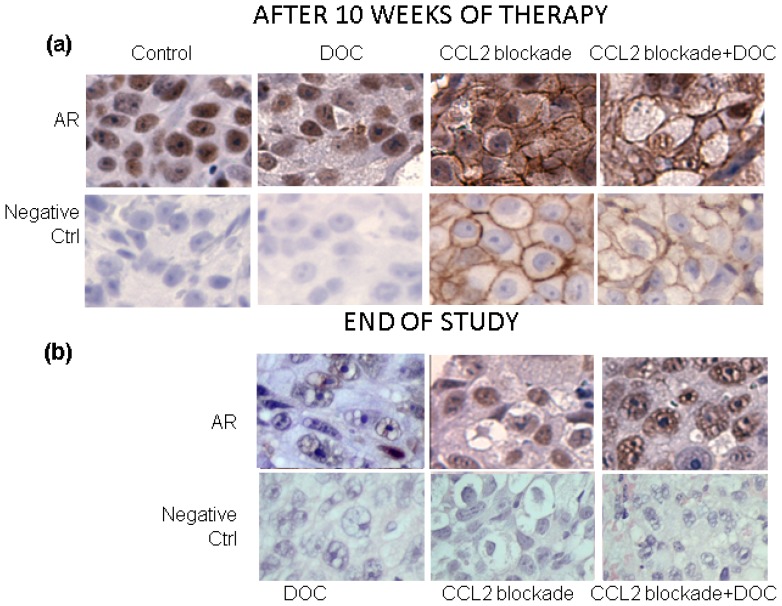
Effects of treatment on cell morphology and AR immunoreactivity. (**a**) C4-2B cells in the bone marrow are minimally differentiated cells organized in solid sheets. Cytologically, these tumor cells show high mitotic rate, moderate nucleus:cytoplasm ratio (N/C ratio) and prominent nucleoli. Most of the tumor cells exhibit moderate to intense nuclear AR immunoreactivity. Docetaxel treatment altered the tumor cells, resulting in slight variability in size and shape and decreased percentage and intensity of AR nuclear immunoreactivity. CCL2 blockade caused cytoplasmic vacuolar changes of tumor cells with abundant, transparent cytoplasm and nuclei compressed eccentrically by the large cytoplasmic vacuole. Weak AR nuclear immunoreactivity was detected in the nuclei. Some immunoreactivity was also detected on the cell membrane. We hypothesize that this membrane staining could result from cross reactivity of the treatment antibodies with the detection antibodies. The most pronounced morphological changes were caused by the combination of CCL2 blockade and docetaxel. Severe vacuolar change of the cytoplasm compressed the nuclei into crescent shapes. Low expression AR nuclear immunoreactivity is present in the tumor cells; (**b**) Nine weeks after treatment cessation, C4-2B cells treated with docetaxel exhibit “foamy”-looking cytoplasm and nuclei, and further loss of nuclear AR is dramatic in this group. Tumor cells treated with CCL2 blockade show vacuolar changes in the cytoplasm, with AR immunoreactivity mostly located in the nuclei with less intense staining in comparison to the cells at the end of the treatment. Membrane immunoreactivity is not present. Pronounced changes are evident in tumors from the combination group. Vacuolar change of the cytoplasm and foamy change of the nuclei are the main features of the tumor cells. AR immunoreactivity is located in the nuclei with moderate expression in more than 90% of the tumor cell population.

CCL2 blockade resulted in significant decreases in serum prostate-specific antigen (PSA) levels over the control animals at weeks 7–10 after the beginning of the treatment, lowering PSA levels in the treated animals at week 10 to 28.9% ± 2.6% (*p* = 0.0038) of control animals (Figure 1A,B). We chose to combine animals treated with placebo (group 1) with those treated with control antibody (group 2) as a single control group for our statistical analyses, because our results showed that treatment with control antibody did not cause significant differences in PSA levels in comparison with untreated animals (*p* = 0.13–0.95). Treatment with docetaxel also resulted in significantly lower PSA levels when compared to the control group 1 week after the beginning of the treatment, resulting in decreased PSA levels at week 10 to 44.6% ± 5.9% (*p* = 0.024) of control animals (Figure 1A,B). CCL2 blockade combined with docetaxel treatment resulted in significantly lowered PSA levels throughout the treatment period, with decreases in PSA levels at week 10 to 18.2% ± 2.1% of the control group (*p* = 0.0010). CCL2 blockade was more effective in decreasing PSA than docetaxel alone; moreover, CCL2 blockade combined with docetaxel treatment resulted in even larger decreases in PSA in comparison to either CCL2 blockade or docetaxel alone (*p* = 0.0027 and *p* = 0.0002, respectively; Figure 1A,B). The inhibition of tumor progression is also clearly indicated by decreased levels of Ki67 in tumors of mice treated with CCL2 blockade (see below). To determine whether tumor suppression persisted after the discontinuation of the therapy, a subset of animals from each group was followed for an additional nine weeks. At the end of the study, PSA levels of animals treated with CCL2 blockade alone and in combination with docetaxel were significantly lower when compared to those of control animals, resulting in a 64% and 83% decrease, respectively (Figure 1C,D). PSA levels of animals treated with docetaxel were also lower *vs.* those of control animals (inhibition of ~25%) but this decrease did not reach significance. Importantly, PSA levels at euthanasia in animals treated with CCL2 blockade and docetaxel were significantly lower than those of animals treated with docetaxel alone (23.5% ± 5.7%, *p* = 0.0028). The comparison of the follow up between CCL2 blockade and the combination therapy showed a decrease of ~50% in the combination therapy group, but these results did not reach significance because of the large variation of PSA levels. A survival analysis demonstrated significant increases in survival when CCL2 blockade was used alone (*p* = 0.0011) as well as in combination with docetaxel (*p* < 0.0001) over control or docetaxel-treated animals (Figure 1F). However, despite no observed progression based on PSA levels and altered morphology of the tumors that were treated with CCL2 blockade, the Ki67 staining analysis showed that while Ki67 was decreased during the treatment phase, at the end of study follow up there was increased positivity *vs.* control tumors and tumors undergoing treatment (Figure 1E). This result may indicate that treatment did not fully eradicate malignant cells and treatment resistant cells are beginning to proliferate. AR staining of tumors showed decreased nuclear AR after treatment with CCL2 blockade and, most importantly, the morphology of the treated cells is clearly altered by the treatments (Figure 2). These changes indicate negative effects of the treatments on tumor cells; severe vacuolar changes of the cytoplasm and compressed, crescent shaped nuclei are caused by CCL2 blockade.

### 2.2. Effects of CCL2 Blockade on Tumor-Bearing Bone

**Figure 3:**
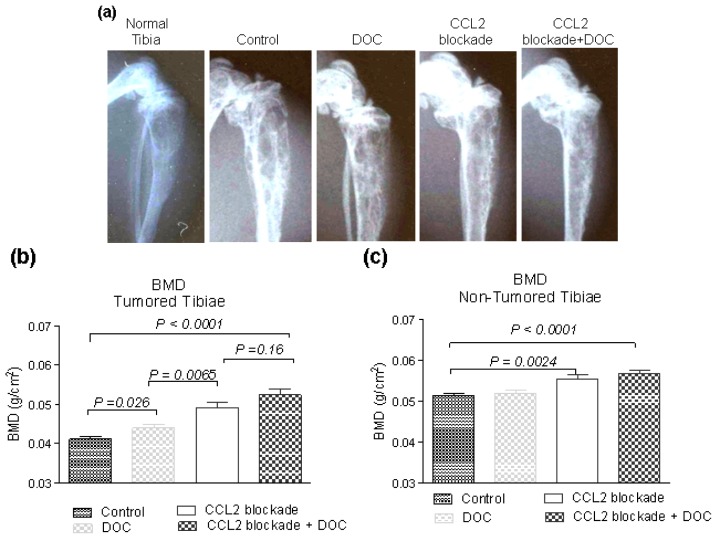
Effects of treatment on bone mineral density (**a**) Representative example radiographs of the tumored tibiae are shown. C4-2B cell growth in the tibiae results in mixed lytic/blastic lesions with expansion of the tibiae. CCL2 blockade decreased changes in the bone caused by the tumors; (**b**) C4-2B cells growing untreated in tibiae typically result in decreases in BMD. All the treatments examined here significantly attenuate these decreases in BMD, with more pronounced effects detected in the groups receiving CCL2 blockade alone or in combination; (**c**) Administration of CCL2 blockade causes significant increases in BMD of normal bone as demonstrated by measurements of BMD in contralateral non-tumored tibiae.

Since CCL2 signaling is important in bone turnover [28], we evaluated the effects of its inhibition on the bone/tumor microenvironment. We focused on whether the blockade of CCL2 signaling inhibits bone destruction and/or bone formation associated with tumor growth. Similar to serum PSA, no differences in BMD were observed in tumored tibiae of animals treated with the control Ab *vs.* vehicle-treated animals (0.043 ± 0.001 g/cm^2^*vs.* 0.040 ± 0.001 g/cm^2^; *p* = 0.074); therefore we combined measurements from the two groups into one control group. Representative examples of X-ray images of control and treated tibiae are shown in Figure 3a. CCL2 blockade alone resulted in a 20.8% ± 3.7% increase in BMD of tumored tibiae over control group tibiae (*p* = 0.0024), treatment with docetaxel alone resulted in a significant 7.8% ± 2.6% increase in BMD of tumored tibiae compared to the control tumored tibiae (*p* = 0.026), and CCL2 blockade in combination with docetaxel resulted in a 28.6% ± 3.9% increase of BMD over the control (*p* < 0.0001). CCL2 blockade resulted in a 12.0% ± 3.4% increase over the docetaxel-treated group (*p =* 0.0065) and combination therapy resulted in 19.2% ± 3.6% increase over the docetaxel alone group (*p* < 0.0001) (Figure 3b). These results show that blocking CCL2 increases BMD in tumor-bearing bone, consistent with its role in bone turnover [28].

### 2.3. Effects of CCL2 Blockade on Normal Bone

Androgen suppression therapy, used as a treatment for advanced and recurrent PCa, causes decreases in BMD and shorter times to skeletal related events. Since CCL2 signaling plays an important role in healthy bone biology, we also set out to determine the effects of CCL2 blockade on normal bone. The BMDs of the non-tumor-bearing contralateral tibiae of animals on CCL2 blockade (single agent or in combination with docetaxel) were significantly higher than BMDs of control and docetaxel-treated animals (Figure 3c), and animals treated with CCL2 blockade exhibited a trend towards increased whole body BMD *vs.* BMD of animals that did not receive CCL2 blockade (data not shown). The difference in BMD of non-tumored tibiae between the groups receiving the mixture of Abs alone *vs.* in combination with docetaxel was not statistically significant (*p* = 0.16), confirming that these changes in BMD were most likely caused by CCL2 blockade. The existence of the systemic effects of CCL2 blockade on bone was also supported by decreased levels of serum Ca^2+^ in the animals treated with CCL2 blockade in comparison to animals that were not treated with CCL2 blockade (11.6 ± 0.5, and 13.3 ± 0.4 respectively, *p* = 0.014) Taken together with the BMD increases seen in tumor-bearing tibiae, these results demonstrate that CCL2 blockade acts in a systemic fashion to affect bone biology throughout the tumor-bearing host.

### 2.4. Effects of CCL2 Blockade on Bodyweight

**Figure 4:**
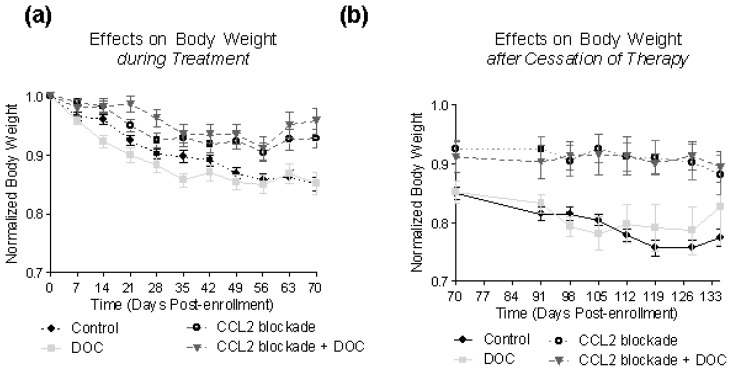
Effects of treatment on body weight. (**a**) Growth of C4-2B cells in tibiae causes weight loss in the control animals, an effect which is abrogated by blockade of CCL2 signaling. Docetaxel does not inhibit this weight loss, despite inhibiting tumor growth; (**b**) Even after treatment was stopped, those animals originally receiving CCL2 blockade had significantly higher body weights compared to control animals or animals treated with docetaxel.

A large percentage of patients with advanced PCa suffer from cachexia as a consequence of their disease and treatment. Therefore, when evaluating efficacy and potential clinical usefulness of new agents it is important to monitor any effects of the treatment on body weight. Our results show that C4-2B cells growing in the bone of control animals resulted in weight loss of the animals, and that CCL2 blockade attenuated this weight loss during and even after the cessation of therapy (Figure 4a,b). Similarly, CCL2 blockade as a single agent or in combination with docetaxel significantly increased body weight compared to docetaxel-only treated animals (*p* < 0.001). Docetaxel as a single agent did not alter body weight in comparison to untreated animals during or after therapy.

### 2.5. Implications

Despite the recent progress in the treatment of advanced metastatic PCa, bone metastasis is still a major problem and new treatment modalities are needed to increase survival and quality of life. In our study we focused on investigation of the efficacy of neutralizing CCL2 and its combination with docetaxel on PCa growth in bone. Docetaxel is a standard treatment for advanced PCa, and it has been shown that docetaxel treatment causes increases in CCL2 expression, while knockdown of CCL2 increases docetaxel efficacy, suggesting a possible mechanism of docetaxel resistance [29]. Our results clearly show significant improvement in serum PSA, BMD, and body weight of the experimental animals receiving the CCL2 blockade as a single agent, and, importantly, even greater beneficial effects when CCL2 blockade is used in combination with docetaxel. While data on the tumor inhibitory effects of anti-CCL2 antibodies and their combination with docetaxel on PC-3 cells has been published previously [10,11], our results show more pronounced effects. Moreover, our results show sustained inhibitory effects for a prolonged time after the cessation of therapy, whereas previous work indicated that tumor regression achieved via CCL2 blockade in combination with docetaxel could be maintained only with continuation of anti-CCL2 therapy following termination of docetaxel, and that tumor growth was quickly reestablished once CCL2 blockade was discontinued. These previous studies used only antibodies to inhibit human and mouse CCL2, and so some of these differences may result from our inclusion of a neutralizing antibody to mouse MCP-5, another agonist of CCR2. This comparison of our results and the published data clearly suggest that to achieve optimal efficacy it may be crucial to inhibit any and all activation of the CCL2 receptor, CCR2. Furthermore, we used C4-2B cells, which elicit a mixed osteoblastic/lytic reaction more representative of PCa in patients, instead of the more aggressive and osteolytic PC-3 cells.

Despite no significant increases in PSA and tumor volume at the end of the study, we detected increased Ki67 positivity at this time point. We hypothesize that because of the highly adaptable nature of metastatic PCa, it is most likely that the resurgence of Ki67 staining detected six weeks after the cessation of the treatment is indicative of the presence of treatment-resistant cells and that potentially if the tumors were followed further we might detect recurrence. Therefore despite the sustained inhibition following cessation of therapy, this treatment still did not lead to eradication of the entire tumor and is therefore unlikely be curative. However, the residual tumors 10 weeks after the end of treatment showed significantly altered morphology consistent with cellular distress, confirming that the anti-CCL2 treatment had, if not fully eliminated tumor cells, at least induced cellular changes that were persistent after the end of therapy. It is also possible that increased apoptosis or senescence is involved in tumor suppression at the end of the study which counteracts the increased Ki67 positivity in the previously treated tumors, though we have not explored this possibility in this work.

The importance of the tumor microenvironment in cancer progression has been clearly documented, and it is well accepted that to improve treatment outcomes agents are needed that modify this microenvironment in addition to tumors themselves. CCL2 blockade holds promise as such a treatment, and our results show alterations in tumor-bearing as well as normal bone. The tumor growth suppression detected might be a combination of not only direct effects on tumor cells but also effects on osteoclasts. Inhibition of bone lysis by CCL2 blockade could contribute to an attenuation of tumor growth and also systemically increase bone mass. This is important for patients with advanced CRPC suffering from age-induced bone loss, tumor-induced bone loss, and treatment-induced bone loss. Therefore our results indicate that CCL2 blockade is a treatment that would be efficacious not only against advanced PCa but also improve overall bone health in patients.

Because of promising preclinical results seen when targeting CCL2 signaling in tumor and bone, CCL2 blockade by neutralizing antibody (CNTO 888) was tested in a clinical phase 2 trial [30]. Unfortunately, the results were not as successful as anticipated: no significant inhibition of tumor progression was detected. Levels of free CCL2 decreased only transiently following the first dose, and then the treatment was unable to durably suppress rising free serum CCL2 levels. The apparent feedback loop leading to elevated CCL2 in response to the blockade might be responsible for the lack of any detectable effects on tumor progression. There were also several other concerns raised such as low *in vivo* binding affinity of the antibody which could potentially release bound CCL2, and the profound prior treatments of enrolled patients. Unfortunately, this is one of many new agents that show efficacy in a preclinical setting and then fall short of achieving therapeutic end points in human patients. In this case, issues that could help explain the disappointment of the clinical trial were recognized and this information, along with the new preclinical data, could be used in the design of new trials enrolling patients earlier during disease progression, using higher doses of the agent, or using new combinations of therapies.

## 3. Experimental Section

### 3.1. Animal Experiments

All animal procedures were performed in compliance with the University of Washington Institutional Animal Care and Use Committee and NIH guidelines. 105 SCID beige mice (Charles Rivers Laboratories, Wilmington, MA, USA) were injected intra-tibially with C4-2B cells (a castration-resistant subline of LNCaP cells [31]) as described previously [32,33]. Blood samples were drawn weekly for determination of serum prostate-specific antigen (PSA) levels (IMx Total PSA Assay, Abbott Laboratories, Abbott Park, IL, USA), which were used to evaluate tumor growth. All therapeutic antibodies were provided by Janssen Research and Development and docetaxel was purchased (LC Laboratories, Woburn, MA, USA). Four weeks after tumor-cell injection when tumors were established (PSA serum levels > 0.6 ng/mL), animals were randomized into five groups: Group 1: Vehicle, (750 μL PBS intraperitoneally (IP)) twice weekly, and 400 μL 0.9% saline IP once every other week (EOW) (*n* = 18); Group 2: Control antibody (Ab), (750 μL Control Ab, 30 mg/kg) IP twice weekly, and 400 μL 0.9% saline IP EOW (*n* = 15); Group 3: CCL2 blockade, mixture of antibodies against human CCL2, murine CCL2, and murine MCP-5 (750 μL pooled antibodies (30 mg/kg, 10 mg/kg each antibody)) IP twice weekly, and 400 μL 0.9% saline IP once EOW (*n* = 19); Group 4: Docetaxel, (400 μL docetaxel, 10 mg/kg) IP once EOW, and 750 μL Control Ab (30 mg/kg) IP twice weekly; and Group 5) CCL2 blockade + docetaxel (750 μL pooled antibodies (30 mg/kg)) IP twice weekly, and 400 μL docetaxel (10 mg/kg) IP EOW (*n* = 19). In patients, CCL2 produced by tumor and host cells is of human origin, while in our model CCL2 produced by tumor cells is human and CCL2 produced by the host microenvironment is a murine homolog. In addition, both mouse and human CCL2 can bind and activate human and mouse CCR2 receptors [34]. Therefore, to mimic the situation in patients we used a mixture of antibodies to inhibit both CCL2 produced by the microenvironment (murine homolog, Ab C1142) and tumor-derived CCL2 (human homolog, Ab CNTO 888). Additionally, we included an antibody against MCP-5 (murine CCL12, Ab C1450), a structural and functional homologue of murine CCL2 which also activates CCR2 [35], in order to inhibit all CCR2-dependent signaling. To monitor responses to the treatments, weekly serum PSA levels and body weights were recorded. Animals were treated for ten weeks. At the end of the treatment period, a subset of animals from each group was euthanized, and the remaining animals were monitored for an additional nine weeks. The numbers of animals in each group after treatment cessation were as follows: group 1: *n* = 6; group 2: *n* = 5; group 3: *n* = 8; group 4: *n* = 8; group 5: *n* = 9. Animals were euthanized when they became compromised or serum PSA levels exceeded 50 ng/ml. Radiographs (Faxitron Specimen Radiography System, Model MX-20, Faxitron X-Ray Corporation, Wheeling, IL, USA) and bone mineral density (BMD) measurements (PIXImus Lunar densitometer, GE Healthcare, Waukesha, WI, USA) were recorded prior to euthanasia.

### 3.2. Immunohistochemistry (IHC)

Decalcified and paraffin embedded tissues were used for IHC analysis. Standard IHC protocol with antigen retrieval was used [36]. The antibodies used were anti-androgen receptor monoclonal antibody (1:50, Biogenex, Fremont, CA, USA) and anti-Ki67 monoclonal antibody (1:100, Dako, Carpinteria, CA, USA).

### 3.3. Statistical Analysis

Student *t*-tests and ANOVA were used to compare the effects of CCL2 blockade, both alone and in combination. Results were considered significant for *p* < 0.05.

## 4. Conclusions

CCL2 signaling remains a promising target in advanced prostate cancer. It has shown tumor inhibitory effects in a range of preclinical studies including ours. Equally important, CCL2 blockade significantly increased bone health and improved the body weight of animals receiving treatment. These results together with further research addressing the issues identified in a recent clinical trial would help in designing new trials with the potential to bring this option into the clinic, providing great therapeutic value to men whose options are quite limited. The data we report here give evidence that if the issues identified in the clinical trial can be overcome, improvements in patient outcomes and quality of life can be expected by targeting this important signaling pathway.
